# Psycholinguistic features, design attributes, and respondent-reported cognition predict response time to patient-reported outcome measure items

**DOI:** 10.1007/s11136-021-02778-5

**Published:** 2021-02-08

**Authors:** Matthew L. Cohen, Aaron J. Boulton, Alyssa M. Lanzi, Elyse Sutherland, Rebecca Hunting Pompon

**Affiliations:** 1 Department of Communication Sciences and Disorders, University of Delaware, 100 Discovery Blvd 6th Floor, Newark, DE 19713 USA; 2 Center for Health Assessment Research and Translation, University of Delaware, Newark, DE 19713 USA

**Keywords:** Patient-reported outcomes, Patient-reported outcome measures, Reaction time, Cognitive impairments, Self-report

## Abstract

**Purpose:**

Patient-reported outcome measures (PROMs) vary in their psycholinguistic complexity. This study examined whether response time to PROM items is related to psycholinguistic attributes of the item and/or the self-reported cognitive ability of the respondent.

**Methods:**

Baseline data from Wave 2 of the Quality of Life in Neurological Disorders (Neuro-QoL) development study were reanalyzed. That sample contained 581 adults with neurological disorders and whose self-reported cognitive abilities were quantified by the Neuro-QoL v2.0 Cognitive Function Item Bank. 185 Neuro-QoL items were coded for several psycholinguistic variables and design attributes: number of words and syllables, mean imageability of words, mean word frequency, mean age of word acquisition, and response format (e.g., about symptom frequency or task difficulty). Data were analyzed with linear and generalized linear mixed models.

**Results:**

Main effects models revealed that slower response times were associated with respondents with lower self-reported cognitive abilities and with PROM items that contained more syllables, less imageable (e.g., more abstract) words, and that asked about task difficulty rather than symptom frequency. Interaction effects were found between self-reported cognition and those same PROM attributes such that people with worse self-reported cognitive abilities were disproportionately slow when responding to items that were longer (more syllables), contained less imageable words, and asked about task difficulty.

**Conclusion:**

Completing a PROM requires multiple cognitive skills (e.g., memory, executive functioning) and appraisal processes. Response time is a means of operationalizing the amount or difficulty of cognitive processing, and this report indicates several aspects of PROM design that relate to a measure’s cognitive burden. However, future research with better experimental control is needed.

**Supplementary Information:**

The online version contains supplementary material available at 10.1007/s11136-021-02778-5.

Patient-reported outcomes (PROs), captured with PRO measures (PROMs), play important roles in clinical research and healthcare by permitting the standardized evaluation of health constructs from the perspective of the patient and other stakeholders [1, 2]. PROMs are especially valuable for measuring health constructs that are difficult for a clinician to fully observe, for example, pain, anxiety, depression, social connectedness, and other constructs with a subjective component [3]. PROMs are being used for clinical research and clinical practice with participants/patients of nearly all ages and health conditions, including individuals with cognitive and language challenges [3, 4].

PROMs may be more or less cognitively difficult for patients to complete based on the health construct(s) assessed, as well as the design and phrasing of the PROM. For example, respondents may more easily (quickly) respond to items about concrete physical symptoms than abstract emotional symptoms, about symptoms within the past hour than within the past week, about frequency of a symptom more than degree of impairment because of that symptom, to items with three response options rather than nine, and to items with common words rather than uncommon words. Test developers may follow best practice guidelines and/or qualitative feedback from pilot respondents when deciding on PROM design characteristics and item phrasing [5, 6], but there is often not a strong empirical research literature informing those kinds of design decisions [7]. However, the cognitive demands required for PROM completion is important to consider and study—for all respondents, not just those with cognitive or language limitations—because they may have important implications. For example, a deeper understanding of PROM response processes may lead to a more complete understanding of response error [8, 9], more valid interpretations of PROM scores, and development of PROMs that are more accessible to people with cognitive and language challenges [10].

Completing a PROM requires the respondent to (1) receive (e.g., aurally, visually) and comprehend the question, (2) search for and retrieve relevant information from memory, (3) apply appraisal processes towards a response (e.g., expectations of health, standards of comparison [11, 12]), and (4) elect and communicate (orally or manually) a response [13]. At a minimum, these stages draw on the respondent’s attention, processing speed, auditory-verbal working memory, episodic memory, language comprehension, and executive functioning [3, 14]. Indeed, there is a reasonably sized literature on psychological variables that may associate with survey responses [6, 15, 16], but without as much emphasis on health constructs in particular like what are assessed by PROMs, or with cognitive science methods like examinations of psychophysiological variables or response time [17]. Since they can quantify cognitive effort, these methodologies are potentially useful as a complement to cognitive interviewing, which relies strongly on the patient’s language and metacognitive abilities, and are only able to capture psychological factors within the patient’s awareness.

In the work presented here, we report on a secondary analysis of PROM item response times, which is a proxy variable for psychological effort or difficulty, to test whether time/effort completing a PROM may be associated with psycholinguistic factors, design attributes, and/or cognitive ability of the respondent [7, 18]. Consistent with findings from the more experimental cognitive science literatures (but to our knowledge not yet extended to PROMs), we hypothesize that longer response time to PROM items (i.e., indicative of more cognitive effort) will be associated with respondents with lower self-reported cognitive function and with PROM items that assess abstract (e.g., stigma) rather than concrete health constructs (e.g., mobility) [19], contain low-frequency words [7] or words that are acquired later in development [20]. We further hypothesize that there will be a response time difference associated with whether the PROM item is phrased as a perception-based question (e.g., asking about frequency of symptoms) or an evaluation-based question (e.g., asking about difficulty completing a task), because these may be associated with different cognitive appraisal processes [21]. However, we do not have a direction associated with that hypothesis. To be clear, we do not think that response time necessarily reflects the validity of the response. Rather, we are interpreting response time as a reflection of the amount of processing, or the effort required for that response.

## Methods

### Participants and procedures

The present study used Wave 2 data from the initial development of the Quality of Life in Neurological Disorders (Neuro-QoL) measurement system [22], obtained from a public repository [23]. Whereas the Neuro-QoL Wave 1a (clinical sample) and 1b (general population sample) cohorts were drawn from online respondent pools, this sample consisted of 581 adults with neurological disorders (multiple sclerosis, Parkinson’s disease, epilepsy, stroke, and amyotrophic lateral sclerosis [ALS]) who were recruited from and tested at 1 of 12 academic medical centers in the United States [22]. As described in greater detail in the main Neuro-QoL development papers [22, 24, 25], participants were eligible for study inclusion if they were: (1) at least 18 years of age, (2) English speaking, (3) diagnosed with one of the five neurological conditions studied, and (4) entered the study with a proxy reporter (for individuals with stroke only; e.g., family member, caregiver). Participants were excluded if (1) cognitive impairment precluded their ability to provide informed consent or understand and complete test items, (2) their seizures were non-epileptic (participants with epilepsy only), or (3) they were not community-dwelling (participants with stroke only). Participants were assessed up to three times; for the present analyses, baseline data were used. Participants completed items within the Assessment Center platform, which recorded their response time [26]. Additional information regarding the Wave 2 sample can be found in Gershon et al. [22] and the Neuro-QoL Technical Report [25] available at www.healthmeasures.net.

### Study measures and variables

#### Outcome variable: Neuro-QoL item response times

The outcome variable for analyses was the response time associated with completing each Neuro-QoL item, between the presentation of the PROM item and the participant’s selection of a response. This time was captured by the Assessment Center platform [26], rounded to the nearest second, and contained in the public Wave 2 dataset [23]. Neuro-QoL comprises 11 item response theory [IRT]-calibrated item banks that assess domains of physical, mental, and social health in the context of neurological conditions. Items use a five-category response format and ask either what Schwartz and Rapkin [21] would call *perception-based questions* about how often specific symptoms occur (*Never/Rarely/Sometimes/Often/Always*) or *evaluation-based questions* about perceived difficulty performing a task (*None/A Little/Somewhat/A lot/Cannot do*).

#### Person-level predictors: patient-reported cognitive function and neurological condition

The Neuro-QoL Item Bank v2.0—Cognitive Function measure consisted of 28 items addressing respondents’ perceived difficulty in completing various cognitive tasks. Scores are provided on a T-metric (*M* = 50, SD = 10), with higher scores reflecting higher levels of perceived cognitive function. In addition to cognitive function, neurological condition was included as a person-level covariate in all models as four dummy-coded variables (reference condition: epilepsy).

#### Item-level predictors: psycholinguistic characteristics

Item-level predictors included psycholinguistic variables extracted from the 185 Neuro-QoL items that were administered in Wave 2 [22, 24]. A full list of item IDs can be found in the online supplementary material. For each Neuro-QoL item, the following variables were coded independently by one of three research assistants. Each research assistant reviewed each psycholinguistic variable and associated database (below), explored example coded items, and practiced coding items until criterion was achieved (100% accuracy on 15 training items). The following psycholinguistic variables were selected because experimental studies from the cognitive science literature have indicated their association with word-processing time.

##### Mean word frequency

The frequency that a word occurs within a given language is associated with how richly represented that word is within the readers’ lexical networks. Infrequent words in the English language (e.g., *platypus*) are less strongly represented and take longer to recognize and respond to than more frequent words (e.g., *dog*) [27]. The mean word frequency was computed for each Neuro-QoL item by entering each word in the item into the SubtlexUS database, adding the frequencies, and dividing by the number of words. The SubtlexUS database is based on subtitles from television and film, and contains over 51 million word instances from over 8000 sources [28].

##### Mean age of word acquisition

Age of word acquisition (AoA) is often operationalized as the age of development at which a person is typically exposed to that word/concept, and is closely tied to but distinct from word frequency [29]. Words that are acquired later in development are processed and responded to more slowly [20]. Mean AoA was computed for each Neuro-QoL item by extracting the AoA for each word from the normative data reported by Kuperman et al. [30], adding the values, and dividing by the number of words in the item.

##### Mean word imageability

Imageability is the “extent to which the referent of a word evokes a mental image [p. 824]” [31]. For example, “dog” evokes a mental image more readily than “justice.” Highly imageable words are associated with shorter processing time than less imageable words [32]. Mean word imageability was computed for each Neuro-QoL item by extracting the imageability for each item from MRC Psycholinguistic database [33].

##### Response format

As mentioned above, Neuro-QoL items, and the items from many other modern PRO measurement systems, are generally perception-based questions about frequency of symptoms or evaluation-based questions about perceived difficulty completing a task. These kinds of questions may involve different cognitive appraisal processes, and associated with different kinds of error [21]. This study coded whether each Neuro-QoL item asked a question about frequency or difficulty.

##### Numbers of words and syllables in the Neuro-QoL item

To minimize the influence of PROM item length on other predictors of response times, we counted the number of words and syllables of each PROM item and included these data in analyses.

### Data analysis

#### Approach

Effects of the item and person-level predictors on Neuro-QoL item response times were analyzed using mixed-effects models [34, 35] to account for two grouping factors in the dataset: persons and items. In the models, persons and items were specified as *crossed-classified* factors because every participant completed every item, thus precluding a hierarchical arrangement of the two factors. That is, items could be considered nested within individuals just as easily as individuals could be considered nested within items.

#### Non-normal response time distribution and outliers

**Fig. 1:**
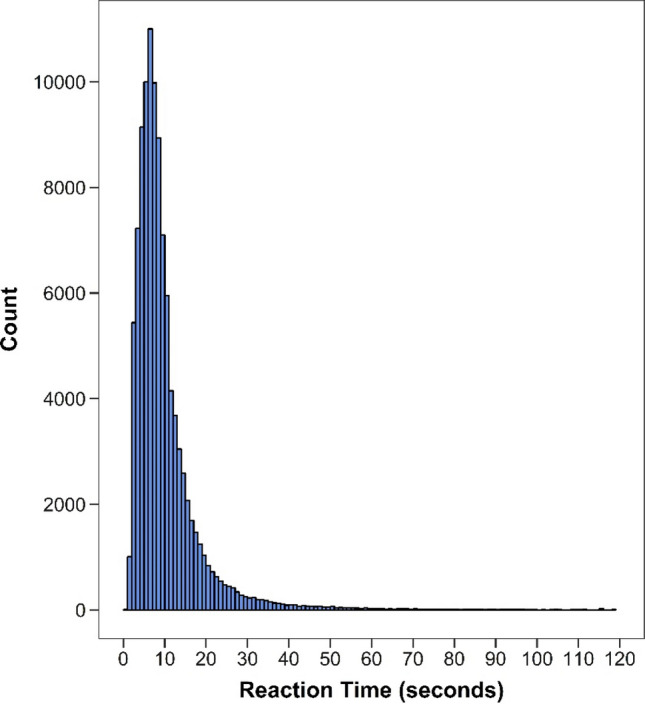
Neuro-QoL item response times. Response times for 177 Neuro-QoL items in the Wave 2 clinical sample (*N* = 577). Approximately 0.25% of recorded response time observations were > 120 s and not shown in the figure

As is common in chronometric research, the distribution of response times consisted of nonnegative values, exhibited severe positive skew, and potentially contained outliers (Fig. 1).

In accordance with the exploratory nature of this study and the need to appropriately analyze the response time distribution, we examined multiple mixed-effects model specifications. Our overarching goal was to build a mixed-effects model that satisfied statistical assumptions to the greatest extent possible while retaining interpretability; in doing so, we aimed in particular for consistency of results across different specifications. These included (a) a linear mixed-effects model (LMM), (b) a LMM with log-transformed response times, and (c) 6 Generalized linear mixed-effects models (GLMMs). More information about the selection, specification, and estimation of each model can be found in the supplementary material.

With regard to outliers, in Fig. 1, approximately 0.25% of the response time observations exceeded the 2-min range of the *x*-axis and are not shown. The maximum response time was 1106 s (~ 18 min), an implausibly high value. A likely explanation for high response time values is that the participant was distracted or took a break during data collection. Unfortunately, it was neither possible to confirm the validity of such data points, nor determine a single cutoff value that separated plausible from implausible values. As a result, multiple cut points were used to determine whether model results were sensitive to trimming a certain portion of the upper tail of the response time distribution prior to model estimation. Specifically, models were estimated using an untrimmed outcome as well as three outcomes trimmed above the 95th, 97th, and 99th percentiles, which correspond to 24, 31, and 52 s, respectively. Cook’s distance, a measure of influence, was also computed for upper-level units to determine whether any single person or item had a considerable impact on model estimates. At the low end of the response time distribution, we removed observations less than half a second (*n* = 11, 0.01%) from the dataset.

#### Model specification, estimation, and evaluation

All models were estimated in R (version 3.6.2, [36]) using the *lme4* package (version 1.1-21). For each type of model and trimming level combination, model building proceeded as follows. First, a main effects model was estimated, in which all item-level and person-level predictors were included. Statistical significance of main effects was determined by inspecting 95% profile likelihood confidence intervals. To test for potential moderation effects of cognitive function on item-level characteristics, interaction terms were entered into the main effects model and tested separately. A final “full” model was then estimated, including all significant terms retained from the previous step. Maximum likelihood estimation was used for all analyses. Due to missing data, the analyzed dataset contained 577 individuals, 177 items, and 100,660 individual item administrations.

## Results

### Data description

Demographic characteristics of the Neuro-QoL Wave 2 clinical sample are provided in Table 1. Results from mixed model analyses were similar across different model specifications and trimming levels; therefore, unless otherwise noted, for the remainder of the article, we report results from the LMM and 99th percentile-trimmed data, which represents a range of plausible response times (1–52 s per PROM item). Results from other analyses can be found in the online supplement.

**Table 1:** Demographic characteristics of Neuro-QoL Wave 2 clinical sample (*N* = 581)

Age (M, SD)	55.21 (14.31)
Sex (%)	
Female	54
Male	46
Race (%)	
White	87
Black/African–American	12
American Indian/Alaskan Native	2
Asian	2
Native Hawaiian/Pacific Islander	0
Occupation (%)	
Homemaker	8
Unemployed	9
Retired	30
Disability	34
Leave of absence	1
Full-time employed	21
Part-time employed	10
Full-time student	1
Marital status (%)	
Married	62
Divorced	11
Widowed	5
Living with someone	5
Separated	2
Never married	16
Income (%)	
> $20,000	16
$20,000–$49,000	35
$50,000–$99,000	28
> $100,000	21
Education (%)	
Some high school or less	3
High school or equivalent	19
Some college	29
College degree	29
Advanced degree	20
Neurological condition (%)	
ALS	14
Epilepsy	20
Multiple sclerosis	28
Parkinson’s	21
Stroke	17

These data were also reported in Gershon et al. [22] and are reprinted here with permission from the copyright holder

Univariate descriptive statistics for item response times, item psycholinguistic characteristics, and cognitive function are provided in Table 2. Bivariate descriptive statistics (correlations) for the predictor variables are provided in Table 3. As expected, the correlation between the number of words and syllables was high (*r* = .91); therefore, only number of syllables was included in the analysis models. All other variables correlated in the small-to-moderate range (*r* = − .44 to .30).

**Table 2:** Descriptive statistics for 99th percentile-trimmed dataset

Variable	*N*	*M*	SD	Median	Min	Max	Skew	Kurtosis
Item psycholinguistic characteristics								
Words	177	8.16	3.88	8	3	26	1.12	2.18
Syllables	177	11.94	6.08	11	3	42	1.25	2.95
Avg. word frequency	177	387,539.08	177,384.88	388,259	10,707	742,078	− 0.05	− 0.58
Avg. age of acquisition	177	4.56	0.83	4.42	2.91	7.21	0.93	0.70
Avg. imageability	177	341.61	60.51	337	179	489	0.30	0.03
Cognitive function scores								
Neuro-QoL cognitive function	577	50.01	9.82	49.7	17.9	69.9	− 0.14	− 0.09
Response times (in s)								
Overall	99,646	9.03	6.84	7	1	52	2.40	8.00
Condition								
ALS	12,911	6.68	5.95	5	1	52	3.13	13.71
Epilepsy	20,690	8.47	6.67	7	1	52	2.43	8.40
MS	27,928	9.97	7.02	8	1	52	2.37	7.26
Parkinson’s	20,944	8.23	6.03	7	1	52	2.66	10.67
Stroke	17,173	10.91	7.50	9	1	52	2.08	5.96
Item bank								
Anxiety	4567	8.48	6.38	7	1	52	2.73	10.89
Ability to participate in SRA	4542	9.53	7.03	8	1	52	2.31	7.51
Cognitive function	22,689	10.42	7.77	8	1	52	2.05	5.51
Depression	4565	7.14	5.42	6	1	52	3.14	14.66
Emot. and behav. dys	10,269	7.51	5.32	6	1	52	2.78	12.40
Fatigue	10,828	8.06	5.69	7	1	52	2.55	10.16
Lower ext.—mobility	9718	11.45	8.17	9	1	52	1.89	4.56
Pos. affect and well being	5120	8.47	6.45	7	1	52	2.37	7.92
Sleep disturbance	11,394	8.26	5.98	7	1	52	2.65	10.84
Stigma	4571	8.26	5.90	7	1	52	2.53	9.34
Upper ext.—fine motor/ADL	11,383	8.60	6.70	7	1	52	2.57	8.96

Values based on 1% trimmed sample (i.e., scores retained at or below 99th percentile)

**Table 3:** Bivariate correlations between item-level predictor variables

	Words	Syllables	Avg. word frequency	Avg. age of acquisition	Avg. imageability
Item psycholinguistic characteristics					
Words					
Syllables	0.91 [0.89, 0.93]				
Average word frequency	− 0.15 [− 0.29, − 0.01]	− 0.28 [− 0.41, − 0.14]			
Average age of acquisition	− 0.12 [− 0.26, − 0.03]	0.10 [− 0.04, 0.24]	− 0.13 [− 0.27, 0.01]		
Average imageability	− 0.42 [− 0.53, − 0.29]	− 0.47 [− 0.58, − 0.35]	0.30 [0.17, 0.43]	− 0.05 [− 0.19, 0.10]	
Response format (difficulty vs. frequency)	0.04 [− 0.11, 0.19]	0.05 [− 0.09, 0.20]	− 0.44 [− 0.55, − 0.31]	0.13 [− 0.01, 0.28]	− 0.06 [− 0.21, 0.09]

Correlations based on 1% trimmed sample (i.e., scores retained at or below 99th percentile)

Correlation coefficients and 95% confidence intervals based on Fisher’s *z* transformation

### Main effects model results

Results for the main effects LMM based on the 99th percentile-trimmed dataset are shown in Table 4. Neuro-QoL v2.0—Cognitive Function scores were negatively associated with item response times such that after controlling for item characteristics and neurological condition, respondents reporting higher levels of cognitive impairment required more time to complete items. As for the item characteristic variables, the number of syllables per item and response format (difficulty vs. frequency) were positively associated with item response times, that is, respondents took more time responding to items that contained more syllables and used a difficulty (vs. frequency) response format, after controlling for other item characteristics, cognitive function, and condition.

**Table 4:** LMM results for 99th percentile-trimmed sample

Model parameters	Main effects only		Full model	
	Est.	CI	Est.	CI
Fixed effects				
Intercept	**7**.**86**	[7.17, 8.55]	**7**.**86**	[7.17, 8.55]
Syllables	**0**.**88**	[0.62, 1.13]	**0**.**88**	[0.63, 1.13]
Word frequency	− 0.22	[− 0.48, 0.05]	-0.22	[− 0.48, 0.05]
Age of acquisition	− 0.06	[− 0.28, 0.16]	− 0.06	[− 0.28, 0.16]
Imageability	0.13	[− 0.12, 0.39]	0.13	[− 0.12, 0.39]
Response format	**1**.**35**	[0.83, 1.87]	**1**.**35**	[0.83, 1.87]
Cognitive function	− **0**.**75**	[− 1.05, − 0.44]	− **0**.**69**	[− 1.00, − 0.39]
ALS (ref = epilepsy)	− 0.91	[− 1.98, 0.15]	− 0.92	[− 1.98, 0.15]
MS (ref = epilepsy)	**1**.**62**	[0.80, 2.45]	**1**.**62**	[0.80, 2.45]
Parkinson’s (ref = epilepsy)	− 0.05	[− 0.94, 0.84]	− 0.05	[− 0.94, 0.84]
Stroke (ref = epilepsy)	**2**.**58**	[1.65, 3.50]	**2**.**58**	[1.65, 3.50]
Syllables × cognitive function			− **0**.**13**	[− 0.17, − 0.09]
Imageability × cognitive function			**0**.**09**	[0.05, 0.12]
Response format × cognitive function			− **0**.**17**	[− 0.24, − 0.10]
Random effects				
Individual	3.46	[3.26, 3.67]	3.46	[3.27, 3.67]
Item	1.46	[1.32, 1.63]	1.50	[1.32, 1.63]
Residual	5.42	[5.40, 5.45]	5.42	[5.40, 5.44]

Coefficients in boldface significant at *p* < .05

### Interaction effects model results

**Fig. 2:**
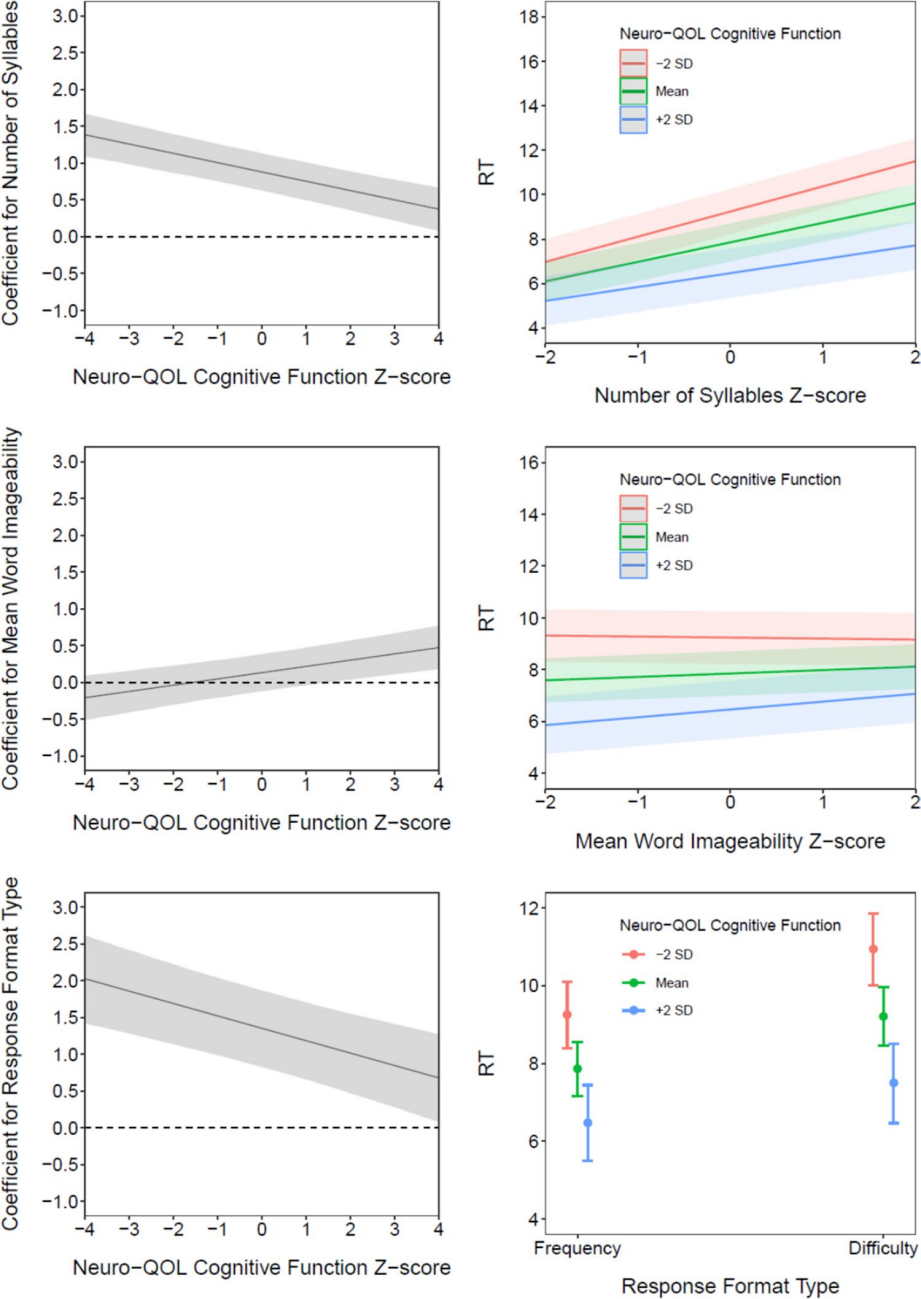
Conditional effects, simple intercepts and slopes. Estimated regression coefficients for three item-level predictors (number of syllables, imageability, response format), conditional on the moderator variable (cognitive function), are shown in the left-hand column. Increased cognitive function was associated with decreased (toward zero) effects for number of syllables and response format, whereas the effect of imageability was increased (away from zero). Associated simple intercepts and slopes—regression lines between each predictor and response time (RT) and plotted at different values of cognitive function (mean and ± 2 standard deviations)—are shown in the right-hand column. These plots show how response times increase with increasing numbers of syllables and when using the difficulty vs. frequency response format, and how these increases are augmented at higher levels of cognitive function. Conversely, for imageability, the positive effect only emerges at higher levels of cognitive function, though the effect is confounded with number of syllables (see Fig. 3)

Significant interaction effects were found between self-reported cognitive function and three item-level predictors: number of syllables, imageability, and response format. Coefficient estimates for these terms are provided in Table 4. Plots of conditional effects, which show how coefficients for the item-level predictors vary as a function of cognitive function, are shown in the left-hand column of Fig. 2. Simple intercepts and slopes are shown in the right-hand column; the regression lines in these plots show the relation between the item-level predictors and response time at three levels of cognitive function (− 2 SD, mean, + 2 SD). There was a significant interaction between the number of syllables in an item and self-reported cognitive function. As shown in Fig. 2, the positive association between the number of syllables and response time was stronger (i.e., further from zero) for respondents with lower levels of self-reported cognitive function.

**Fig. 3:**
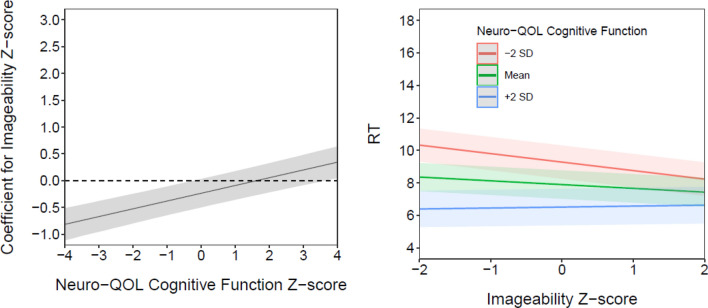
Conditional effects and simple intercepts and slopes for imageability, when number of words is omitted from model. Estimated regression coefficients for imageability, conditional on the moderator variable (cognitive function) and without the number of words included in the model, are shown on the left. Associated simple intercepts and slopes, plotted at the mean and ± 2 standard deviations and without the number of words included in the model, are shown on the right-hand column

There was also a significant interaction effect between imageability and self-reported cognitive function such that the effect of imageability on response time was larger for individuals with higher levels of cognitive function. This effect is apparent in Fig. 2, as the trend line of the coefficient becomes significantly different from zero in the positive direction approximately 2 SDs above the mean. However, this moderation effect appears to be confounded with the number of syllables per item. When the number of syllables per item is removed from the model, the trend line representing the conditional effect is shifted downward (Fig. 3), such that it becomes significantly different from zero in the negative direction for individuals approximately 1 SD or more below the mean of cognitive function. This appears due to a negative statistical suppression effect, such that the direction and statistical significance of the association between imageability and response time—that is, the main effect—differs depending on whether the number of syllables is included in the model. When the number of syllables is removed from the main effects model, imageability has a negative and statistically significant effect on response time, such that items with lower imageability ratings are associated with slower response times (controlling for other predictors). Although the slope of the conditional effect line does not change, the downward shift that results from the suppression complicates interpretation of the interaction effect. This can be seen in Figs. 2 and 3 in the simple intercept/slopes plots as the “fanning” effect created by the interaction changes directions depending on whether the number of syllables in an item is also modeled. Inspection of partial correlations among the study variables and additional exploratory analyses suggested that other main and interaction effects were not similarly impacted.

Finally, there was a significant interaction effect between response format and self-reported cognitive function. As shown in Fig. 2, the conditional effect line, which represents the difference in response times between the two types of response formats, is further from zero in the positive direction for individuals with lower levels of self-reported cognitive function. That is, the effect of response format was larger for individuals with lower self-reported cognitive function scores.

## Discussion

Completing a PROM draws on multiple cognitive skills to support the respondent as they receive and comprehend the question, search for and retrieve relevant information from memory, apply appraisal processes towards a response, and select and communicate a response. To more fully understand variables that contribute to cognitive effort completing a PROM, the work presented here was a secondary analysis of data that aimed to evaluate whether a respondent’s self-reported cognitive abilities and attributes of a PROM item was associated with their response time to that item.

Consistent with our hypothesis, lower self-reported cognitive abilities were indeed associated with slower response times. However, this finding should be interpreted alongside several considerations. First, items that contributed to the Neuro-QoL v2.0 Cognition T score assessed multiple abilities (attention, memory, etc.) so we are not able to discern which abilities were most strongly related to response time. Second, previous research has indicated that self-reported cognition is only moderately correlated with performance-based cognitive test scores, and equally correlated with symptoms of depression [37]. Finally, we cannot form conclusions about the causes of cognitive limitations (e.g., neurological condition), or how they contribute to response time. Individuals in this sample with stroke or MS displayed the slowest response times to PROM items compared with individuals with ALS, PD, or epilepsy. However, these participants were not recruited to address condition-specific hypotheses. The finding that people with stroke and MS were slowest may have resulted from the particular people with those conditions who were recruited, and we are not interpreting group response times as generalizable findings. Future research should investigate at a more granular level and with performance-based cognitive tests how specific conditions and cognitive substrates relate to PROM appraisal processes and effort.

Respondents took longer to respond to PROM items that contained more syllables. While this main effect is a relatively superficial finding, a more interesting effect is that people with lower self-reported cognitive abilities were disproportionately slowed by longer items. This response may result from limits in verbal working memory capacity, which can limit sentence processing depending on level of attention to the item or perceived item demands [38, 39]. Notably, we did not examine sublexical (e.g., syllable frequency) or syntactic complexity contributions to response time, which may have influenced our findings about item length and response time.

Items with perceptual phrasing (e.g., about symptom frequency) versus evaluative phrasing (e.g., about difficulty completing a task) also associated with response times. Although we did not have an a priori hypothesis about the direction of this effect, our findings were consistent with the model of Schwartz and Rapkin [21]: evaluation-based questions are often associated with longer response times because these items involve more idiosyncratic appraisal processes. Indeed, respondents who reported cognitive limitations were disproportionately slowed by evaluation-based questions, which further supports the hypothesis that evaluation-based questions require more processing. For example, unlike questions about frequency (e.g., “how *often* do you take your medications as prescribed”?), questions about difficulty (“how *difficult* is it for you to take your medications as prescribed?) may require the respondent to generate ideas about what is meant by *difficult* (e.g., mental effort to remember medications, frequency of errors, and number of times corrected by a care partner), and to recall personal experiences over the response period. Our results indicate that people with perceived cognitive limitations are disproportionately slow when completing those mental processes.

Consistent with our hypothesis, word imageability was associated with response time such that PROM items with less imageable (more abstract) words were associated with slower responses. However, this main effect was only significant when number of syllables was removed from the model. We suspect this was due to a negative statistical suppression effect; imageability and number of syllables were moderately correlated, and logically more abstract concepts may require more words/syllables to describe. When the number of syllables were removed from the model, imageability was a significant predictor of response time. Furthermore, word imageability significantly interacted with self-reported cognition (when the number of syllables was removed from the model) such that people reporting cognitive limitations were disproportionately slow responding to PROMs with low imageable words. This coheres with other studies indicating that more abstract words/concepts are less robustly encoded within and between lexical networks, and therefore, require greater strength of activation for comprehension and subsequent response [40]. Our data indicate that people reporting cognitive limitations were disproportionately slow when completing items containing abstract concepts. However, more research is needed with more rigorous, experimental designs.

Against our hypotheses, word frequency and AoA were not associated with response time. Neuro-QoL items were by design written below a 6th grade reading level, as defined by the Lexile Analyzer, based on word frequency and syntax complexity [41], which may have restricted the range of word frequencies contained in Neuro-QoL items, as well as AoA, which is strongly correlated with word frequency [42].

Overall, our data indicate that lower self-reported cognitive abilities and some attributes of PROM design are associated with response time, which indicates the amount of processing required for that response. To be clear, we do not think that response time necessarily reflects the validity of the response; within the range of plausible responses analyzed here, we assume all PROM item responses are equally valid. Rather, we are interpreting response time as a reflection of the amount of processing or effort required for that response. However, questions and concerns have been raised about the cognitive burden of some PROMs and the reliability of PROM responses from people with cognitive and language disorders [3, 14]. For example, Carlozzi et al. reported that lower cognitive abilities were associated with lower internal reliability of PROM items completed by people with Huntington’s disease [43]. PROMs that are too cognitively or linguistically difficult for a respondent could result in unreliable score estimates (and therefore, more difficulty detecting change), invalid score interpretation, subsequent inappropriate clinical action, wasted time, and poor rapport.

The work presented here indicates that the cognitive effort required to complete PROMs may be reduced by relatively simple design changes, for example, using concise phrasing, more highly imageable (e.g., concrete rather than abstract) words, and composing items to focus on frequency rather than difficulty. A good example of this is the work of Hunting Pompon et al. [10], who modified the Perceived Stress Scale [44] to be more accessible to people with post-stroke aphasia by simplifying the phrasing of the scale’s instructions and items, altering the scale’s format to include more white space to lessen visual distraction, and including an associated graphic element to the Likert scale response options. There are likely other design factors that could be associated with response time that deserve future study, for example, syntactic complexity, phonotactic probability, emotional evocativeness, and graphic response options. Future studies of PROM response times should take place under controlled, experimental conditions, and capture more granular differences in response time. Ultimately, research on the cognitive demands of PROM completion is potentially important for all respondents so that the field may have a more complete understanding of response error [8, 9], more valid interpretations of PROM scores, and development of PROMs that are more accessible to people with cognitive and language challenges [10].

## Supplementary Information

Below is the link to the electronic supplementary material.

Supplementary file1 (DOCX 45 KB)

## Data Availability

Data were obtained from the Healthmeasures Dataverse [23].

## Code availability

N/A.

## References

[1] Bartlett SJ, Ahmed S (2017). Montreal Accord on patient-reported outcomes (PROs) use series—Paper 1: Introduction. Journal of Clinical Epidemiology.

[2] Bingham CO, Noonan VK, Auger C, Feldman DE, Ahmed S, Bartlett SJ (2017). Montreal Accord on patient-reported outcomes (PROs) use series—Paper 4: Patient-reported outcomes can inform clinical decision making in chronic care. Journal of Clinical Epidemiology.

[3] Cohen ML, Hula WD (2020). Patient-reported outcomes and evidence-based practice in speech-language pathology. American Journal of Speech-Language Pathology.

[4] Yorkston K, Baylor C (2019). Patient-reported outcomes measures: An introduction for clinicians. Perspectives of the ASHA Special Interest Groups.

[5] Dillman, D. A., Smyth, J. D., & Christian, L. M. (2014). How to write open- and close-ended questions. In *Internet, phone, mail, and mixed-mode surveys: The tailored design method* (4th ed.). Hoboken: Wiley.

[6] Schwarz N (2001). Asking questions about behavior: Cognition, communication, and questionnaire construction. The American Journal of Evaluation.

[7] Lenzner T, Kaczmirek L, Lenzner A (2010). Cognitive burden of survey questions and response times: A psycholinguistic experiment. ACP Applied Cognitive Psychology.

[8] Knäuper B, Belli RF, Hill DH, Herzog AR (1997). Question difficulty and respondents’ cognitive ability: The effect on data quality. Journal of Official Statisticsm.

[9] Velez P, Ashworth SD (2007). The impact of item readability on the endorsement of the midpoint response in surveys. Survey Research Methods.

[10] Hunting Pompon R, Amtmann D, Bombardier C, Kendall D (2018). Modifying and validating a measure of chronic stress for people with aphasia. Journal of Speech Language and Hearing Research.

[11] Rapkin BD, Schwartz CE (2004). Toward a theoretical model of quality-of-life appraisal: Implications of findings from studies of response shift. Health and Quality of Life Outcomes.

[12] Rapkin BD, Schwartz CE (2019). Advancing quality-of-life research by deepening our understanding of response shift: A unifying theory of appraisal. Quality of Life Research.

[13] Jobe JB (2003). Cognitive psychology and self-reports: Models and methods. Quality of Life Research: An International Journal of Quality of Life Aspects of Treatment, Care and Rehabilitation.

[14] Barrett AM (2010). Rose-colored answers: Neuropsychological deficits and patient-reported outcomes after stroke. Behavioural Neurology.

[15] Tourangeau R, Rips LJ, Rasinski KA (2000). The psychology of survey response.

[16] Schwarz N (1999). Self-reports: How the questions shape the answers. American Psychologist.

[17] Baldwin P, Yaneva V, Mee J, Clauser BE, Ha LA (2020). Using natural language processing to predict item response times and improve test construction. Journal of Educational Measurement.

[18] Swanson DB, Case SM, Ripkey DR, Clauser BE, Holtman MC (2001). Relationships among item characteristics, examine characteristics, and response times on USMLE Step 1. Academic Medicine.

[19] Xiao X, Zhao D, Zhang Q, Guo C (2012). Retrieval of concrete words involves more contextual information than abstract words: Multiple components for the concreteness effect. Brain and Language.

[20] Catling JC, Dent K, Johnston RA, Balding R (2010). Age of acquisition, word frequency, and picture–word interference. Quarterly Journal of Experimental Psychology (2006).

[21] Schwartz CE, Rapkin BD (2004). Reconsidering the psychometrics of quality of life assessment in light of response shift and appraisal. Health and Quality of Life Outcomes.

[22] Gershon RC, Lai JS, Bode R, Choi S, Moy C, Bleck T (2012). Neuro-QOL: Quality of life item banks for adults with neurological disorders: Item development and calibrations based upon clinical and general population testing. Quality of Life Research: An International Journal of Quality of Life Aspects of Treatment, Care and Rehabilitation.

[23] Cella D (2017). NeuroQOL clinical validation study (aka Wave II). Harvard Dataverse.

[24] Cella D, Lai J-S, Nowinski CJ, Victorson D, Peterman A, Miller D (2012). Neuro-QOL: Brief measures of health-related quality of life for clinical research in neurology. Neurology.

[25] *Neuro-QoL Technical Report*. (2015). Retrieved from www.neuroqol.org.

[26] Gershon R, Rothrock NE, Hanrahan RT, Jansky LJ, Harniss M, Riley W (2010). The development of a clinical outcomes survey research application: Assessment CenterSM. Quality of Life Research.

[27] Balota DA, Chumbley JI (1984). Are lexical decisions a good measure of lexical access? The role of word frequency in the neglected decision stage. Journal of Experimental Psychology. Human Perception and Performance.

[28] Brysbaert M, New B (2009). Moving beyond Kucera and Francis: A critical evaluation of current word frequency norms and the introduction of a new and improved word frequency measure for American English. Behavior Research Methods.

[29] Brysbaert M, Ghyselinck M (2006). The effect of age of acquisition: Partly frequency related, partly frequency independent. Visual Cognition.

[30] Kuperman V, Stadthagen-Gonzalez H, Brysbaert M (2012). Age-of-acquisition ratings for 30,000 English words. Behavior Research Methods.

[31] de Groot AMB (1989). Representational aspects of word imageability and word frequency as assessed through word association. Journal of Experimental Psychology: Learning, Memory, and Cognition.

[32] Schwanenflugel PJ, Harnishfeger KK, Stowe RW (1988). Context availability and lexical decisions for abstract and concrete words. Journal of Memory and Language.

[33] Coltheart M (1981). The MRC psycholinguistic database. The Quarterly Journal of Experimental Psychology Section A.

[34] Pinheiro J, Bates D (2000). Mixed-effects models in S and S-PLUS.

[35] Baayen RH, Davidson DJ, Bates DM (2008). Mixed-effects modeling with crossed random effects for subjects and items. Journal of Memory and Language.

[36] R Core Team. (n.d.). *R: A language and environment for statistical computing*. Vienna, Austria: R Foundation for Statistical Computing. Retrieved from https://www.r-project.org/.

[37] Lai J-S, Goodnight S, Downing NR, Ready RE, Paulsen JS, Kratz AL (2018). Evaluating cognition in individuals with Huntington disease: Neuro-QoL cognitive functioning measures. Quality of Life Research.

[38] Caplan D, Waters GS (1999). Verbal working memory and sentence comprehension. Behavioral and Brain Sciences.

[39] McVay JC, Kane MJ (2012). Why does working memory capacity predict variation in reading comprehension? On the influence of mind wandering and executive attention. Journal of Experimental Psychology: General.

[40] Plaut DC, Shallice T (1993). Deep dyslexia: A case study of connectionist neuropsychology. Cognitive Neuropsychology.

[41] DeWalt DA, Rothrock N, Yount S, Stone AA, PROMIS Cooperative Group (2007). Evaluation of item candidates: The PROMIS qualitative item review. Medical Care.

[42] Raling R, Hanne S, Schroder A, Kessler C, Wartenburger I (2017). Judging the animacy of words: The influence of typicality and age of acquisition in a semantic decision task. Quarterly Journal of Experimental Psychology (2006).

[43] Carlozzi NE, Schilling S, Kratz AL, Paulsen JS, Frank S, Stout JC (2018). Understanding patient-reported outcome measures in Huntington disease: At what point is cognitive impairment related to poor measurement reliability?. Quality of Life Research.

[44] Cohen S, Janicki-Deverts D (2012). Who’s stressed? Distributions of psychological stress in the United States in probability samples from 1983, 2006, and 20091: PSYCHOLOGICAL STRESS IN THE U.S. Journal of Applied Social Psychology.

