# Supplementary material for: Psycholinguistic features, design attributes, and respondent-reported cognition predict response time to patient-reported outcome measure items
Source: Qual Life Res. 2021 Feb 8;30(6):1693–704. doi: 10.1007/s11136-021-02778-5 (PMC8178143; doi:10.1007/s11136-021-02778-5)
Supplement: Supplementary file 1 — Supplementary file1 (DOCX 45 KB) [file 11136_2021_2778_MOESM1_ESM.docx]

**Supplementary Material to**

**“Psycholinguistic Features, Design Attributes, and Respondent-Reported Cognition**

**Predict Response Time to Patient-Reported Outcome Measure Items”**

**Neuro-QOL Item Banks and Items**

In the table below, Neuro-QOL item banks and items included in the analyses reported in the main paper are provided. The psycholinguistic features of 185 items were extracted. Of these, 8 items representing a single domain (Satisfaction with Social Roles and Activities) were excluded from analyses because the response format for the items could not be categorized as representing difficulty or frequency. Items were drawn from a combination of entire item banks, item bank short forms, and fixed-length measures, as described in the table.

| Table S1. Neuro-QOL Item Banks and Items Included in Analysis | | |
| --- | --- | --- |
| Item Bank | Form | Item IDs |
| Anxiety | SF | NQANX07 NQANX09 NQANX20 NQANX22 NQANX23 NQANX26 NQANX27 NQANX28 |
| Ability to Participate in Social Roles and Activities | SF | NQPRF01 NQPRF03 NQPRF08 NQPRF09 NQPRF17 NQPRF26 NQPRF32 NQPRF34 |
| Communication | FLF | NQCOG01 NQCOG02 NQCOG03 NQCOG04 NQCOG08 NQCOG10 NQCOG11 NQCOG61 |
| Cognitive Function | IB^a^ | NQCOG46 NQCOG53 NQCOG64 NQCOG65 NQCOG66 NQCOG67 NQCOG68 NQCOG69 NQCOG70 NQCOG72 NQCOG73 NQCOG74 NQCOG75 NQCOG77 NQCOG80 NQCOG83 NQCOG84 NQCOG86 NQCOG15 NQCOG16 NQCOG17 NQCOG22 NQCOG24 NQCOG25 NQCOG26 NQCOG27 NQCOG28 NQCOG31 NQCOG38 NQCOG39 NQCOG40 NQCOG51 |
| Depression | SF | NQDEP02 NQDEP07 NQDEP13 NQDEP19 NQDEP21 NQDEP23 NQDEP24 NQDEP27 |
| Emotional and Behavioral Dyscontrol | IB | NQPER01 NQPER02 NQPER05 NQPER06 NQPER07 NQPER08 NQPER09 NQPER10 NQPER11 NQPER12 NQPER13 NQPER14 NQPER15 NQPER16 NQPER17 NQPER18 NQPER19 NQPER20 |
| Fatigue | IB | NQFTG01 NQFTG02 NQFTG03 NQFTG04 NQFTG05 NQFTG06 NQFTG07 NQFTG08 NQFTG09 NQFTG10 NQFTG11 NQFTG12 NQFTG13 NQFTG14 NQFTG15 NQFTG16 NQFTG17 NQFTG18 NQFTG20 |
| Lower Extremity Function – Mobility | IB | NQMOB01 NQMOB03 NQMOB04 NQMOB06 NQMOB08 NQMOB09 NQMOB11 NQMOB16 NQMOB17 NQMOB21 NQMOB23 NQMOB25 NQMOB26 NQMOB28 NQMOB30 NQMOB31 NQMOB32 NQMOB33 NQMOB37 |
| Positive Affect and Well-Being | SF | NQPPF07 NQPPF12 NQPPF14 NQPPF15 NQPPF16 NQPPF17 NQPPF19 NQPPF20 NQPPF22 |
| Satisfaction with Social Roles and Activities | SF | NQSAT03 NQSAT11 NQSAT14 NQSAT23 NQSAT32 NQSAT33 NQSAT46 NQSAT47 |
| Sleep Disturbance | FLF^b^ | NQSLP01 NQSLP02 NQSLP03 NQSLP04 NQSLP05 NQSLP06 NQSLP07 NQSLP08 NQSLP09 NQSLP10 NQSLP11 NQSLP12 NQSLP13 NQSLP14 NQSLP15 NQSLP16 NQSLP17 NQSLP18 NQSLP19 NQSLP20 |
| Stigma | SF | NQSTG01 NQSTG02 NQSTG04 NQSTG05 NQSTG08 NQSTG16 NQSTG17 NQSTG21 |
| Upper Extremity Function – Fine Motor/ADL | IB | NQUEX03 NQUEX04 NQUEX05 NQUEX06 NQUEX15 NQUEX19 NQUEX20 NQUEX23 NQUEX28 NQUEX29 NQUEX30 NQUEX31 NQUEX32 NQUEX33 NQUEX36 NQUEX37 NQUEX38 NQUEX39 NQUEX41 NQUEX44 |

Note. ADL = Activities of Daily Living; SF = Short Form; IB = Item Bank; FLF = Fixed-length Form

^a^ Items comprise the Neuro-QOL v1.0 Executive Function and Neuro-QOL v1.0 General Concerns items banks. Additionally, and uncalibrated item (NQCOG51) from the original Executive Function pool was included in analyses.

^b^ Items comprise the entire original item pool, which includes 12 uncalibrated items and 8 calibrated items.

**Model Specification, Estimation, and Evaluation**

As noted in the main article, the item response times consisted of positive values that followed a non-normal, positively skewed distribution. Such distributions are unlikely to satisfy the statistical assumptions underlying *linear* mixed-effects models (LMM) (e.g., normally distributed, homoscedastic residuals), leading to considerable debate over best analytical practices. One widely used approach involves transforming the response time variable to better match normal theory-based assumptions, for instance, by applying a logarithmic or inverse transformation (or, more generally, a power transformation) prior to model estimation. Although transformed data will often result in residuals that better align with LMM assumptions, interpretation of coefficients can often become complicated as the nonlinear nature of common transformations alters the relation between the outcome variable and model predictors.[1] As a result, some have argued that analysis on the raw response time metric may be preferred, as linear mixed-effects models are somewhat robust to violations of select model assumptions.[2] Additionally, there may be theoretical reasons to prefer the raw time metric, depending on the cognitive process or processes under study.[1] In recent years, generalized linear mixed models (GLMMs), which provide greater selection in terms of outcome distribution shapes and functional relations between predictors and outcomes, have also been advocated as an optimal compromise between the competing demands of theoretical soundness and statistical fidelity.[1] GLMMs are often more complicated to estimate, however, and may not be computationally tractable in all modeling situations.[3]

This debate over best practices remains unresolved, and because of the exploratory nature of the study, it was decided to analyze the response times using multiple mixed-effects model specifications and examine the consistency of results across the different specifications. Therefore, the data were analyzed by fitting a LMM, a LMM with a log-transformed outcome variable, and several GLMMs. To specify a GLMM, a distribution must be chosen for the outcome variable as well as a *link function* which connects the outcome variable to the set of predictors on the right-hand side of the model equation, called the *linear predictor* in GLMM parlance. Following recent tutorials,[1] a total of six GLMM specifications were used, resulting from all combinations between two outcome distributions (Gamma distribution, inverse Gaussian distribution) and three link functions (identity, logarithmic, and inverse link functions). The Gamma and inverse Gaussian distributions are well-suited to modeling skewed continuous variables with positive values, and are commonly combined with the listed link functions. All LMM and GLMM models were estimated in R (version 3.6.2) using the *lme4* package (version 1.1-21). As noted in the main paper, each model specification was crossed with the four levels of trimming (95%, 97%, 99%, no trimming).

**Additional Analysis Results**

Abbreviated results from all analyses are shown in Tables S2 and S3. Only model coefficients are reported. Results are reported for three specifications: LMM, a LMM with a log-transformed outcome, and a Gamma GLMM with a log link function. All other GLMM specifications failed to converge, regardless of dataset trimming level. Results from the main effects models across model specification and trimming level are shown in Table S2; results from the full model, including the interaction terms, are shown in Table S3. Overall, results were largely similar across models and trimming levels. For higher trimming levels, coefficients slightly attenuated toward zero regardless of direction, although patterns of statistical significance remained consistent with a small number of exceptions; for instance, the dummy-coded effect quantifying the difference between participants with ALS vs. Epilepsy. One notable exception, however, was the interaction effect between response format and cognitive function. This interaction was only significant in three of the four LMM models and one of the Gamma GLMMs. Because the LMM is the most interpretable model specification and was chosen for the final results, the interaction is reported as significant in the main article. Additional research is needed to further examine this effect before strong conclusions can be made.

Table S2 – Additional Main Effects Model Results for Lognormal LMM and Gamma (Log-Link) GLMM

|  | LMM | | | | Lognormal | | | | | Gamma w/Log Link | | | |
| --- | --- | --- | --- | --- | --- | --- | --- | --- | --- | --- | --- | --- | --- |
| Model Parameters | 95 | 97 | 99 | NT | 95 | 97 | 99 | NT^a^ | 95 | | 97 | 99 | NT^a^ |
| Fixed Effects |  |  |  |  |  |  |  |  |  | |  |  |  |
| Intercept | **7.24** | **7.49** | **7.86** | **8.13** | **1.79** | **1.81** | **1.83** | **1.84** | **1.89** | | **1.92** | **1.96** | **2.02** |
| Syllables | **0.70** | **0.77** | **0.88** | **1.04** | **0.09** | **0.09** | **0.09** | **0.10** | **0.08** | | **0.09** | **0.09** | **0.10** |
| Word Frequency | -0.14 | -0.17 | -0.22 | -0.27 | -0.02 | -0.02 | -0.02 | -0.02 | -0.02 | | -0.02 | -0.02 | -0.02 |
| Age of Acquisition | -0.06 | -0.07 | -0.06 | -0.25 | -0.01 | -0.01 | -0.01 | -0.01 | -0.01 | | -0.01 | -0.01 | -0.02 |
| Imageability | 0.07 | 0.08 | 0.13 | 0.33 | 0.01 | 0.01 | 0.01 | 0.01 | 0.01 | | 0.01 | 0.01 | 0.02 |
| Response Format | **0.92** | **1.09** | **1.35** | **3.05** | **0.11** | **0.12** | **0.13** | **0.15** | **0.12** | | **0.13** | **0.14** | **0.20** |
| Cognitive Function | **-0.59** | **-0.66** | **-0.75** | **-0.95** | **-0.08** | **-0.08** | **-0.08** | **-0.09** | **-0.08** | | **-0.08** | **-0.08** | **-0.09** |
| ALS (ref = Epilepsy) | **-0.88** | **-0.93** | -0.91 | -0.61 | **-0.16** | **-0.16** | **-0.16** | **-0.16** | **-0.14** | | **-0.15** | **-0.15** | -0.12 |
| MS (ref = Epilepsy) | **1.38** | **1.47** | **1.62** | **2.45** | **0.23** | **0.23** | **0.24** | **0.24** | **0.20** | | **0.20** | **0.20** | **0.20** |
| Parkinson’s (ref = Epilepsy) | 0.11 | 0.04 | -0.05 | 0.16 | 0.04 | 0.03 | 0.03 | 0.03 | 0.02 | | 0.01 | 0.00 | 0.02 |
| Stroke (ref = Epilepsy) | **2.10** | **2.31** | **2.58** | **3.39** | **0.29** | **0.30** | **0.31** | **0.32** | **0.27** | | **0.28** | **0.29** | **0.31** |
| Random Effects |  |  |  |  |  |  |  |  |  | |  |  |  |
| Individual | 2.71 | 2.99 | 3.46 | 5.99 | 0.40 | 0.41 | 0.43 | 0.43 | 0.20 | | 0.22 | 0.27 | 0.59 |
| Item | 1.04 | 1.19 | 1.46 | 2.30 | 0.13 | 0.13 | 0.14 | 0.15 | 0.08 | | 0.09 | 0.11 | 0.24 |
| Residual | 3.40 | 4.03 | 5.42 | 20.69 | 0.39 | 0.41 | 0.45 | 0.50 | 0.46 | | 0.50 | 0.59 | 1.25 |

*Note*. NT = not trimmed.

Coefficients significant at the p < .05 are highlighted in **bold**. Cells containing results reported in the main manuscript are highlighted in gray.

^a^ A value of 0 is not included in the support for a gamma distribution. As a result, all 0s (e.g., < 1 sec rounded down in the dataset) were excluded from analyses.

Table S3 – Additional Full Model Results for Lognormal LMM and Gamma (Log-Link) GLMM

|  | LMM | | | | Lognormal | | | | | Gamma w/Log Link | | | |
| --- | --- | --- | --- | --- | --- | --- | --- | --- | --- | --- | --- | --- | --- |
| Model Parameters | 95 | 97 | 99 | NT | 95 | 97 | 99 | NT^a^ | 95 | | 97 | 99 | NT^a^ |
| Fixed Effects |  |  |  |  |  |  |  |  |  | |  |  |  |
| Intercept | **7.24** | **7.49** | **7.86** | **8.13** | **1.79** | **1.81** | **1.83** | **1.84** | **1.89** | | **1.92** | **1.96** | **2.02** |
| Syllables | **0.70** | **0.77** | **0.88** | **1.04** | **0.09** | **0.09** | **0.09** | **0.10** | **0.08** | | **0.09** | **0.09** | **0.10** |
| Word Frequency | -0.14 | -0.17 | -0.22 | -0.27 | -0.02 | -0.02 | -0.02 | -0.02 | -0.02 | | -0.02 | -0.02 | -0.02 |
| Age of Acquisition | -0.06 | -0.06 | -0.06 | -0.25 | -0.01 | -0.01 | -0.01 | -0.01 | -0.01 | | -0.01 | -0.01 | -0.02 |
| Imageability | 0.07 | 0.08 | 0.13 | 0.33 | 0.01 | 0.01 | 0.01 | 0.01 | 0.01 | | 0.01 | 0.01 | 0.02 |
| Response Format | **0.92** | **1.09** | **1.35** | **3.05** | **0.11** | **0.12** | **0.13** | **0.15** | **0.12** | | **0.13** | **0.14** | **0.20** |
| Cognitive Function | **-0.56** | **-0.62** | **-0.69** | **-0.92** | **-0.07** | **-0.08** | **-0.08** | **-0.09** | **-0.08** | | **-0.08** | **-0.08** | **-0.10** |
| ALS (ref = Epilepsy) | **-0.88** | **-0.93** | -0.92 | -0.61 | **-0.16** | **-0.16** | **-0.16** | **-0.16** | **-0.14** | | **-0.15** | **-0.15** | **-0.12** |
| MS (ref = Epilepsy) | **1.38** | **1.48** | **1.62** | **2.45** | **0.23** | **0.23** | **0.24** | **0.24** | **0.20** | | **0.20** | **0.20** | **0.20** |
| Parkinson’s (ref = Epilepsy) | 0.11 | 0.04 | -0.05 | 0.16 | 0.04 | 0.03 | 0.03 | 0.03 | 0.02 | | 0.01 | 0.00 | 0.02 |
| Stroke (ref = Epilepsy) | **2.10** | **2.31** | **2.58** | **3.39** | **0.29** | **0.30** | **0.31** | **0.32** | **0.27** | | **0.28** | **0.29** | **0.31** |
|  |  |  |  |  |  |  |  |  |  | |  |  |  |
| Syllables × Cognitive Function | **-0.09** | **-0.10** | **-0.13** | **-0.17** | **-0.01** | **-0.01** | **-0.01** | **-0.01** | **-0.01** | | **-0.01** | **-0.01** | **-0.01** |
| Imageability × Cognitive Function | **0.06** | **0.07** | **0.09** | **0.19** | **0.01** | **0.01** | **0.01** | **0.01** | **0.01** | | **0.01** | **0.01** | **0.02** |
| Response Format × Cognitive Function | **-0.10** | **-0.13** | **-0.17** | **-**0.10 | 0.00 | 0.00 | 0.00 | 0.00 | 0.00 | | 0.00 | 0.00 | **0.02** |
|  |  |  |  |  |  |  |  |  |  | |  |  |  |
| Random Effects |  |  |  |  |  |  |  |  |  | |  |  |  |
| Individual | 2.71 | 2.99 | 3.46 | 5.99 | 0.40 | 0.41 | 0.43 | 0.43 | 0.20 | | 0.22 | 0.27 | 0.59 |
| Item | 1.04 | 1.19 | 1.46 | 2.30 | 0.13 | 0.13 | 0.14 | 0.15 | 0.08 | | 0.09 | 0.11 | 0.24 |
| Residual | 3.40 | 4.03 | 5.42 | 20.69 | 0.39 | 0.41 | 0.45 | 0.51 | 0.46 | | 0.50 | 0.59 | 1.25 |

*Note*. NT = not trimmed.

Coefficients significant at the p < .05 are highlighted in **bold**.

^a^ A value of 0 is not included in the support for a gamma distribution. As a result, all 0s (e.g., < 1 sec rounded down in the dataset) were excluded from analyses. Cells containing results reported in the main manuscript are highlighted in gray.

References

1. Lo, S., & Andrews, S. (2015). To transform or not to transform: Using generalized linear mixed models to analyse reaction time data. Frontiers in psychology, 6, 1171.

2. Maas, C. J., & Hox, J. J. (2004). Robustness issues in multilevel regression analysis. Statistica Neerlandica, 58(2), 127-137.

3. Van Rynald, T. L. (2018). Consequences of Power Transforms in Linear Mixed-effects Models of Chronometric Data*.* The University of North Carolina at Chapel Hill.
